# Potential Environmental and Human Health Risks Caused by Antibiotic-Resistant Bacteria (ARB), Antibiotic Resistance Genes (ARGs) and Emerging Contaminants (ECs) from Municipal Solid Waste (MSW) Landfill

**DOI:** 10.3390/antibiotics10040374

**Published:** 2021-04-01

**Authors:** Uttpal Anand, Bhaskar Reddy, Vipin Kumar Singh, Amit Kishore Singh, Kavindra Kumar Kesari, Pooja Tripathi, Pradeep Kumar, Vijay Tripathi, Jesus Simal-Gandara

**Affiliations:** 1 Department of Molecular and Cellular Engineering, Jacob Institute of Biotechnology and Bioengineering, Sam Higginbottom University of Agriculture, Technology and Sciences, Prayagraj 211007, India; ushuats@gmail.com; 2 Division of Plant Pathology, ICAR-Indian Agricultural Research Institute, Pusa, New Delhi 110012, India; 24breddy@gmail.com; 3 Department of Botany, Institute of Science, Banaras Hindu University, Varanasi 221005, India; vipinks85@gmail.com; 4 Department of Botany, Bhagalpur National College, Tilka Manjhi Bhagalpur University, Bhagalpur 812007, India; kishoreamit03@gmail.com; 5 Department of Applied Physics, School of Science, Aalto University, 00076 Espoo, Finland; kavindra_biotech@yahoo.co.in; 6 Department of Computational Biology and Bioinformatics, Jacob Institute of Biotechnology and Bioengineering, Sam Higginbottom University of Agriculture, Technology and Sciences, Prayagraj 211007, India; pooja.tripathi@shiats.edu.in; 7 Applied Microbiology Laboratory, Department of Forestry, North Eastern Regional Institute of Science and Technology, Nirjuli 791109, India; pkbiotech@gmail.com; 8 Nutrition and Bromatology Group, Department of Analytical and Food Chemistry, Faculty of Food Science and Technology, Ourense Campus, University of Vigo, E-32004 Ourense, Spain

**Keywords:** municipal solid waste (MSW), landfill leachate, antibiotic-resistant bacteria (ARB), antibiotic resistance genes (ARGs), metal resistance genes (MRGs), groundwater, bioaerosol, soil, human health

## Abstract

The disposal of municipal solid waste (MSW) directly at landfills or open dump areas, without segregation and treatment, is a significant concern due to its hazardous contents of antibiotic-resistant bacteria (ARB), antibiotic resistance genes (ARGs), and metal resistance genes (MGEs). The released leachate from landfills greatly effects the soil physicochemical, biological, and groundwater properties associated with agricultural activity and human health. The abundance of ARB, ARGs, and MGEs have been reported worldwide, including MSW landfill sites, animal husbandry, wastewater, groundwater, soil, and aerosol. This review elucidates the occurrence and abundance of ARB, ARGs, and MRGs, which are regarded as emerging contaminants (ECs). Recently, ECs have received global attention because of their prevalence in leachate as a substantial threat to environmental and public health, including an economic burden for developing nations. The present review exclusively discusses the demands to develop a novel eco-friendly management strategy to combat these global issues. This review also gives an intrinsic discussion about the insights of different aspects of environmental and public health concerns caused due to massive leachate generation, the abundance of antibiotics resistance (AR), and the effects of released leachate on the various environmental reservoirs and human health. Furthermore, the current review throws light on the source and fate of different ECs of landfill leachate and their possible impact on the nearby environments (groundwater, surface water, and soil) affecting human health. The present review strongly suggests the demand for future research focuses on the advancement of the removal efficiency of contaminants with the improvement of relevant landfill management to reduce the potential effects of disposable waste. We propose the necessity of the identification and monitoring of potential environmental and human health risks associated with landfill leachate contaminants.

## 1. Introduction

The rapid growth of human populations and economic development and its associated industrialization and urbanization has led to a significant expansion of municipal solid waste (MSW) [1,2,3]. Generally, MSW is made up of domestic, medical, agricultural waste, or any rubbish garbage that is not segregated and are mainly disposed of in the same landfill [4]. The result of this is that most cities worldwide struggle to meet the UN SDGs (United Nations Sustainable Development Goals) of solid waste reduction [5]. The generation of the massive quantities of MSW and its mismanagement is a global challenge and poses environmental risks such as pollution and reduced social wellbeing worldwide [6,7,8]. Presently, MSW generation is approximately two billion tonnes each year globally, of which nearly 33% are not managed by municipal authorities [9]. Moreover, it is estimated that the global MSW generation will massively increase by 3.40 billion tonnes by 2050 [10]. Unfortunately, in most countries, dumpsites are the primary disposal route, with 84% collection efficiency and only 15% recycling efficiency of municipally collected MSW [10]. According to Hoornweg and co-workers [11], it is estimated that 4.3 billion urban populations are produced around 1.42 kg of MSW/person, which is expected to increase to 6.1 million metric tonnes/day production of MSW by the year 2025. Despite years of globalization, landfilling is unfortunately still one of the most common old-fashioned methods that are not perfectly designed to stop contamination in soil and groundwater through toxic leachate percolation. Landfill leachate contains undesirable toxic materials, such as organic pollutants, antibiotics, pharmaceutical and personal care products (PPCPs), and heavy metals, that can be percolating during rainfall through disposable waste and contaminate mainly the soil layers and groundwater [12,13]. All these toxic pollutants are harmful to the survival of aquatic life and the food web that can cause various problems for human health, including genotoxicity and cancer-causing effects. Apart from these chemical contaminants, the presence of microbial contaminants (antibiotic-resistant bacteria (ARB), antibiotic resistance genes (ARGs), MRGs, and pathogenic bacteria) in landfill leachate is another major concern, because it can transmit the ARGs in human pathogens via horizontal gene transfer (HGT) [10,14]. Due to these reasons, the removal of antibiotics and PPCPs from landfill leachate is very crucial to protecting the aquatic environment and transmission of ARGs from the environment to humans [15,16,17]. The decomposition of food scraps and a huge amount of MSW in landfill sites produce greenhouse gases (CH_4_ and CO_2_). These gases have the highest global warming capacity. The incessant breathing of landfill gasses by a human may cause tachycardia, fatigue, nausea, collapse, vomiting, and mortality in the worst cases [18]. In the present time, food waste composting into organic fertilizer is the most widely used alternative to food scrap landfilling worldwide, but it also is important to design new methods to improve the removal of ARGs during composting and reduce the risk of ARG dissemination into the soil environment. The decomposition of food scraps also generated the organic leachate that can significantly change the bacterial community with time and temperature and initiate the growth of pathogenic bacteria such as *Salmonella, Pseudomonas, Enterobacteria*, and *Clostridium perfringens* [19,20]. 

Due to the complexity and limited information about the leachate, it is very difficult to characterize all the possible components that drive the proliferation of human pathogens and other pollutants. Currently, landfill leachate is a serious concern for environmental and global public health, and more attention should be given to deeper insights. Keeping in mind the above, the present review summarizes the challenges associated with landfills and antibiotics in the environment, including resistance. Moreover, the environmental impact of MSW is discussed in detail, focusing on aquatic ecosystems, agriculture, ecosystems, and human health. 

## 2. Antibiotic-Resistant Bacteria (ARB) and Antibiotic Resistance Genes (ARGs) in MSW Landfills 

**Figure 1:**
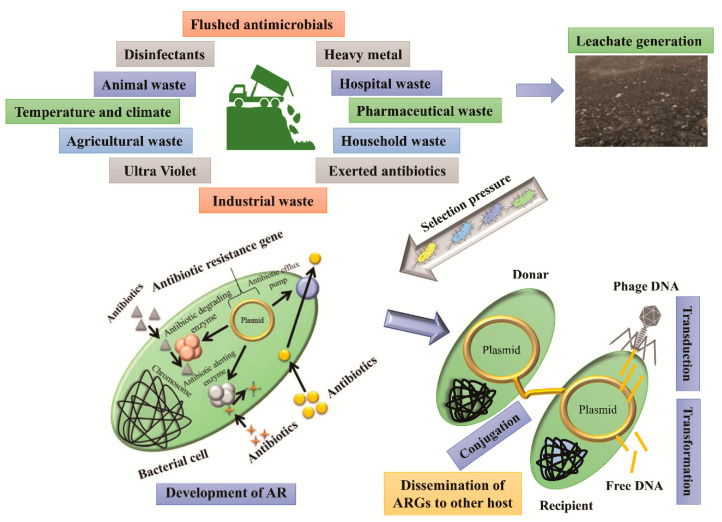
Sources of antibiotics in the landfill system and their mechanisms to adopt an antibiotics resistance (AR) through horizontal gene transfer (HGT). ARGs: antibiotic resistance genes.

Landfills provide a favourable environment to proliferate antimicrobial resistance (AMR) microbes, which further transfer ARGs (horizontal) into outer environment bacterial strains [21,22]. This has several negative effects on public health, wildlife, and the overall environment, such as soil, surface water, and groundwater [23]. Landfill leachate is a complex hazardous liquid that contains antibiotics and toxic organic pollutants, which could promote the evolution of ARB and ARGs in the environment [24,25]. The presence of antibiotic residues in a landfill can generate selected pressure on the environmental bacterial communities and create a reservoir of ARB and ARGs [26,27,28,29]. However, the environmental burden exerted due to the release of ARB and ARGs in various natural environmental compartments, including groundwater and soil, is a very rare and random event. The transmission risk of ARBs from the environment to humans is probably higher than the natural environment. The mechanism of antibiotic adaptation and dissemination is shown in Figure 1. The emergence of ARB in humans can imbalance the intestinal microbiome and proliferate pathogenic bacteria and superbugs, which can cause intestinal imbalance, bowel cancer, colon cancer, and even death due to incurability [30,31,32]. The varieties of complex factors responsible for the dissemination of ARGs include disinfectants, heavy metals, and antibiotics [33,34,35,36]. The overconsumption of antibiotics has led to the development of multidrug-resistant bacteria with HGT via free DNA to the surrounding environments, such as landfill and soil, through donor bacteria by mating, phages, and from dead cells to living cells (Figure 1) [37,38,39,40,41,42]. 

Previous studies have documented that plasmids play a key role in the HGT of ARGs among environmental bacteria [43,44,45]. Notably, the transfer of broad-host-range conjugative self-transmissible plasmids, such as the 60-kb RP4 plasmid (isolated from *Pseudomonas aeruginosa*) that possesses genes for tetracycline resistance, ampicillin resistance, and kanamycin resistance, facilitate the dissemination of ARGs [46]. Although relationships have been found between MSW landfill leachate and antibiotics and the levels of ARGs associated, limited research has been carried out in light of their potential relation at the metagenomic level. Moreover, the composition, toxicity level, diversity, and identification of MGEs and ARGs in MSW landfills are still largely unexplored. This calls for further research, since MSW harbour a great amount of different classes of antibiotics and other anthropogenic compounds such as surfactants, pesticides, and heavy metals that are important to take into account when understanding ARGs in the environment. 

**Figure 2:**
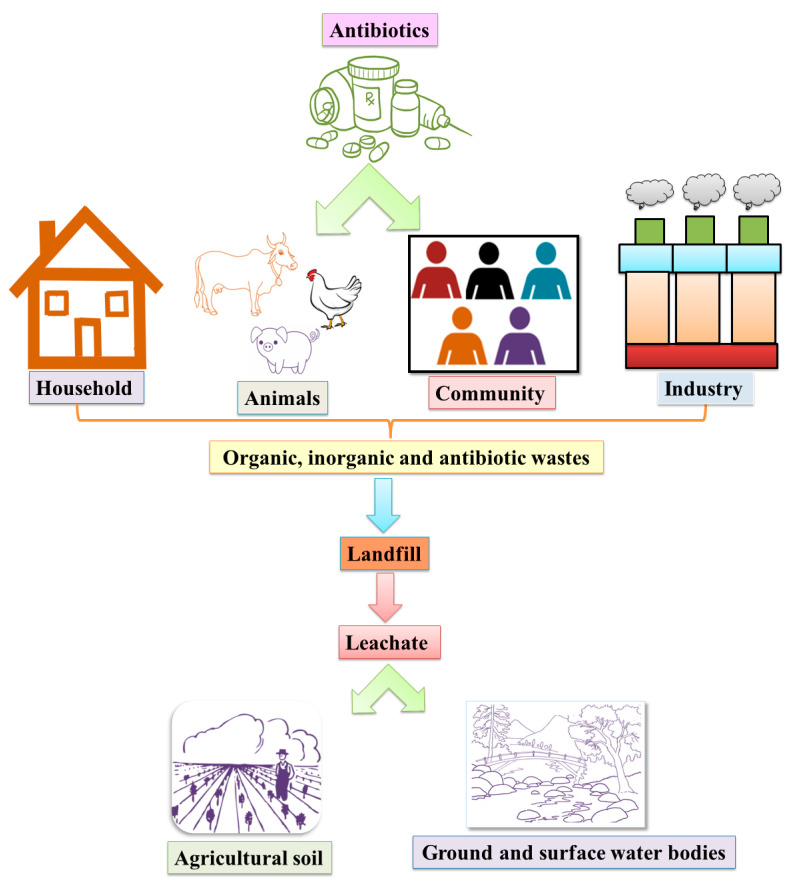
Wastes releases from households, agriculture sites, animal husbandry, and industries accumulate in the municipal solid waste (MSW) landfill, which, finally, affects the surface or groundwater level through leachate percolation. The overall perspective of contaminations through various sources defines the groundwater contaminations in the proposed model.

Threedeach et al. [47] compared the ABR of *Escherichia coli* in leachate samples from anaerobic and semi-aerobic MSW landfill operations, both of which manifest a high resistance to broad classes of antibiotics. The study showed that the presence of antibiotics in leachate can affect the susceptibility of *E. coli* in the landfill. Wang et al. [14] discovered that ARGs are tremendously associated with the bacterial 16S-rRNA gene copies, in 12 landfill leachate samples, which were originated from six different geographic sites in China, with ages varying from five months to eight years. This study revealed that the presence of diverse bacterial populations in landfill leachate was involved in the dissemination of ARGs through HGT. Most significantly, Song et al. [48] reported that antibiotics and ARGs are strongly associated with some of the physiochemical parameters (like nitrate concentrations and the moisture content) of landfill refuge. Furthermore, age, climatic conditions, and quality of the landfill leachate impose a significant impact on the concentrations of heavy metals, microbial diversity composition, antibiotics, and the ARG profiles and compositions in landfill leachate [49,50,51,52]. The microbial taxa also play a major role in ARG distribution in the landfill and nearby environments [53]. Hence, several crucial factors like MGEs, heavy metals, landfill ages, antibiotics, and microbial diversity are important players in the abundance and dissemination of ARGs in landfill leachate associated with agro- and aquaculture [52,54,55,56]. *Enterococcus faecalis* isolated from a contaminated dumpsite showed 100% resistance to chloramphenicol, erythromycin, and tetracycline [57] and showed that the antibiotic and metal resistance genes are frequently carried on the MGEs. A close relationship between metal resistance and AR was found in the landfill of Okhla near New Delhi, where the disposable materials mostly contained pharmaceuticals and industrial wastes [58]. This is therefore likely a reservoir for both ARGs and MRGs. Surprisingly, a significant correlation between the resistance of antibiotics (Kanamycin, monosulphate, tetracycline, and sulfamethoxazole) and heavy metals manganese (Mn) and nickel (Ni) was reported from the lakes and sewages nearby [59]. Overall, this shows that landfills provide a favourable environment for ARB and resistant gene transfer and become a major hotspot for antimicrobial resistance (data presented in Table 1). The overall waste collection in landfill sites from different sources and their roles in the contamination of agricultural soil, surface water, and groundwater are shown in Figure 2. 

## 3. Emerging Contaminants (ECs) in MSW Landfills

Landfill leachate is the reservoir of potentially hazardous contaminants, including organic, inorganic metals, and metalloids. These contaminants belong to a very broad range, and their occurrence (concentration) in the leachate greatly varies in magnitude depending on the landfill’s physical and chemical characteristics [68]. During the rain, these contaminants are released into the nearby soil profiles, groundwater, and surface water, ultimately leading to contaminate natural resources. Under natural conditions, the transport and fate of inorganic contaminants is governed by the properties of contaminants, organic matter content, and sediment characteristics in terms of ion exchange behaviour and mineralogy [69,70]. A plethora of studies has strongly demonstrated that MSW landfill leachate contains high levels of hazardous toxic elements and antibiotics are directly discharged into the MSW landfill, especially in developing countries [71]. It should be noted that the higher concentration of Al (<0.5 mg/L), Fe (<1.0 mg/L), Cu (<1 mg/L), Mg (<200 mg/L), and Zn (<5 mg/L) in the aquatic ecosystem and groundwater are potentially harmful to the human health [72] and have a prolonged association with various diseases such as cardiovascular disease (CD), cancer, Alzheimer disease (AD), Huntington disease (HD), diabetes mellitus (DM), Parkinson disease (PD), etc. [73,74,75]. Currently, various classes of antibiotics are frequently entering the environment through/via wastewater treatment plants (WWTPs), hospital wastewater, the pharmaceutical industry, and landfills [76], massively polluting the overall environment. Hence, antibiotics are considered as one of the important emerging xenobiotic compounds in recent years. Nevertheless, antibiotics are less persistent as compared to metals and persistent organic pollutants in the natural environment, including sulphonamides with a half-life of more than 60 days and even up to 300 days in landfills [52,66].

**Figure 3:**
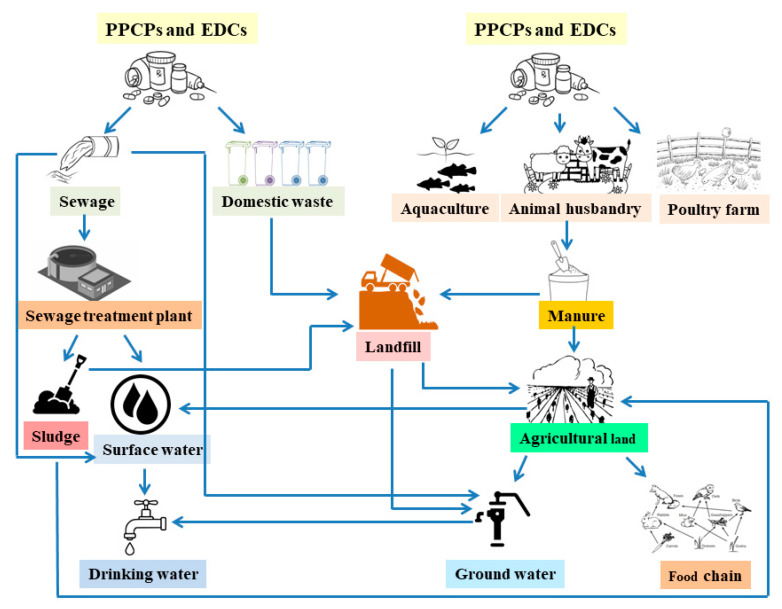
Flow diagram showing the possible pathway for the pharmaceutical and personal care products (PPCPs) and endocrine-disrupting chemicals (EDCs) to enter into landfill sites from different sources (domestic waste, animal husbandry, aquaculture, poultry farm, and sewage) and contaminate the different environmental compartments, such as drinking water, groundwater, agricultural soil, and the food chain.

MSW landfill leachate contains PPCPs, all being endocrine-disrupting chemicals (EDCs) originating from wastewater effluents, hospitals, pharmaceutical industry wastewater, and landfill leaching, as shown in Figure 3 [77,78,79]. According to several studies, PPCPs in the environment have adverse effects on human health and wildlife, such as metabolism dysfunction, muscles locomotion, reproduction, and kidney and gill integrity in fish [25,80,81,82]. Moreover, the presence of PPCPs in the environment potentially influences antibiotic resistance in natural bacterial strains. Table 2 describes the detailed description of PPCPs and ECs detected in various landfill leachate and nearby groundwater reservoirs across the globe.

Nowadays, microplastics have been reported as an emerging contaminant to act as a potential vector of pathogenic bacteria, ARGs, and make up about 20% of the MSW dumped [83,84,85]. MPs have been found in a variety of environments, including landfills, soils, WWTPs, rivers, and oceans [86,87]. The role of MPs in the environment as a source and sink for hazardous chemicals and pathogenic microorganisms is presently a major source of concern, as their bioaccumulation and eventual entry into the food chain represent a global threat [88,89,90]. Plastics or MPs, in particular, can serve as factitious surfaces for microbe colonization and greatly favour the adsorption of other chemicals/pollutants (such as antibiotics, ARGs, EDCs, heavy metals, etc.) that could significantly enhance their negative impact on various environments and humans [91,92,93]. As a result, MPs served as appropriate carriers of these contaminants, altering their environmental transportation strategies and effects in the overall process. A plethora of recent investigation revealed that various microorganisms have been discovered to be closely attached to MPs such as bacteria [94,95], diatoms [96], and fungi [97,98,99]. These associations may also influence the expression of genes involved in the motility and adhesion to surfaces [100]. These microbes are known as the “plastisphere” because of the peculiar existence of the microbial population attached to the MPs or plastics [101].

In light of this, it is very important to remove ECs (PPCPs, EDCs, ARGs, etc.) from landfills to keep aquatic and agricultural ecosystems safe and decrease the transmission of antibiotic resistance from different environments to humans. Most importantly, the landfill sites need particular attention to minimize the threat resulting from the leaching of hazardous PPCP and EDC transport into a nearby aquifer system.

The degradation and removal of most of the ECs mainly performed through biological processes such as anaerobic membrane, aerobic membrane, and anoxic membrane reactors [102,103,104]. However, some ECs (such as PPCPs and antibiotics) are removed through conventional processes (ozonation, centrifugal separation, filtration, flocculation, degritting, screening, coagulation, wetlands treatment, sedimentation, aerobic and anaerobic treatments, and UV photolysis) and nonconventional processes (electrodialysis, precipitation, microfiltration, ultrafiltration, adsorption, oxidation, forward osmosis, solvent extraction, distillation, evaporation, ion exchange, reverse osmosis, electrolysis, and crystallization) [105,106]. For example, xenoestrogens were removed up to 64% by 10 lagoons in a series, 70% by different bioreactors, 80% by two oxidation ditches procedures, and 92% by a conventional activated sludge treatment [107]. The chlorination and solar photolysis methods are very prominent, and strategies are emerging for the removal and degradation of PPCPs from the water [79]. Nevertheless, these methods have the drawbacks of inefficiency and high cost. Furthermore, these methods are ineffective to fully degrade and eliminate some ECs such as clofibric and carbamazepine [108]. Currently, nonconventional methods are extensively used to overcome the shortcomings of conventional methods and to improve the removal capability and performance of ECs. Importantly, the photocatalysts techniques have become well-known in recent years due to their significance in green chemistry [109].

**Table 2:** The detection of pharmaceutical and personal care products (PPCPs), emerging contaminants (ECs), and endocrine-disrupting chemicals (EDCs) in landfill leachate and nearby groundwater environments. MSW: municipal solid waste.

Sr. No.	Landfill/Nearby Sample Collection Station and Country	Sampling Date	Emerging Contaminants (ECs)/Endocrine Disrupting Chemicals (EDCs)/PPCPs	Concentration/Range	References
1	Jiangchungou (JCG) sanitary landfill, City Xi’an, China, 51 samples from 8 locations	August through October 2013	Oxytetracycline (OTC)	100.9 ± 141.81 μg/kg	[48]
			Tetracycline (TC)	63.8 ± 37.7 μg/kg	
			Sulfamethoxazole (SMX)	47.9 ± 8.1 μg/kg	
2	Five USA MSW landfills	Not mentioned	*N,N*-diethyl-m-toluamide (DEET) (organic contaminants)	6900–143,000 ng/L or 6.90–143 μg/L	[15]
			Sucralose (organic contaminants)	<10–621,000 ng/L	
3	Horizontal subsurface flow (HSSF)-constructed wetlands (CWs) system, Singapore	July 2015 to June 2016 at six different sampling points (monthly sampling campaign)	Acetaminophen (ACT)	701–4938 ng/L	[110]
			Bisphenol A (BPA)	138–473,977 ng/L	
			Caffeine (CF)	225–1257 ng/L	
			*N,N*-diethyl-m-toluamide (DEET)	275–2982 ng/L	
			Gemfibrozil (GFZ)	3–46 ng/L	
			Salicylic acid (SA)	196–911 ng/L	
			Sulfamethazine (SMZ)	62–438 ng/L	
4	Leachates from solid waste disposal sites, Japan	Not mentioned	BPA	26,000–8,400,000 ng/L	[111]
5	Leachate was collected from 7 private landfills and 12 municipal landfills sites at various locations of USA	Summer and fall of 2011	Acetophenone	2000–80,000 ng/L	[112]
			Menthol	1600–64,000 ng/L	
			Camphor	400–16,000 ng/L	
			Triclosan	1600–64,000 ng/L	
			Carbaryl	300–12,000 ng/L	
			Pentobarbital	8000–32,000 ng/L	
			beta-Sitosterol	24,000–960,000 ng/L	
			Primidone	16,000–64,000 ng/L	
			4-Nonylphenol	8000–320,000 ng/L	
			Stigmastanol	17,000–680,000 ng/L	
			_D_-Limonene	800–32,000 ng/L	
			*para*-creso	7,020,000 ng/L	
			BPA	6,380,000 ng/L	
6	Landfill reservoir in Shanghai, China	Landfill leachates (November 2014, January, May and October 2015) and treated landfill leachates (January and October 2015)	Phenol	1,550,000 ng/L	[113]
			Diclofenac (DCF)	4810–19,300 ng/L	
			Gemfibrozil (GFZ)	2010–4480 ng/L	
7	Groundwaters (GWs) samples and Besòs River sample, Spain	May 2010, December 2010, May 2011	Salicylic acid (SA)	33.4–620 ng/L	[114]
			Azithromycin	31.5–1620 ng/L	
8	Laogang landfill, Shanghai, China	April and July 2014	Sulfamethoxazole (SMX)	1539.6–8488.0 ng/L	[67]
			Erythromycin (ETM)	1769.5–39,800.5 ng/L	
9	Grindsted Landfill, Japan	Not Mentioned	BPA	1300–17,200,000 ng/L	[115]
10	Laogang landfill, Shanghai, China and livestock wastewater from concentrated animal feeding operations (CAFO) Shanghai, China	November and December 2019	Albendazole (ABZ) in leachates	1.03–61.6 μg/L	[116]
			Albendazole (ABZ) in livestock wastewaters	0.65–2.75 μg/L	
			DEET in landfill leachates	955 ng/L to 58.1 μg/L	
11	Municipal WWTPs (wastewater treatment plants), China	Not mentioned	Anthelmintics: Mebendazole (MEB)	<LOQ (limit of quantification) ~ 223.5 ng/L	[117]
12	Leachate samples from three municipal landfills from Western Sweden (Göteborg area)	March 1996	Di-(2-ethylhexyl) phthalate (DEHP)	97,000–346,000 ng/L	[118]
13	Different MSW landfill locations, Norway	Not mentioned	Pesticides	0.5–110 µg/L	[119]
			Brominated compounds	0.02–11.1 µg/L	
			Polychlorinated biphenyls (PCB)	0.01–3.1 µg/L	
			Mercury (Hg)	0.005–62 µg/L	
14	Landfill, East China (Jinan City, Shandong Province, China)	July and December 2019	Fluoroquinolones, macrolides, and sulfonamides	2.0~5080 ng/L	[120]
15	Four MSW landfills were selected to collect 10 leachate samples in north Italy	Samples were collected from October 2016 to February 2017	Sulfadiazine	2056–2,2102 ng/L	[121]
			Sulfamethoxazole	7978–9,5816 ng/L	
			Sulfadimidine	3898–8450 ng/L	
			Ciprofloxacin	0–434,740 ng/L	
			Enrofloxacin	0–9074 ng/L	
			Erythromycin	8510–252,824 ng/L	
16	European Union (EU) landfill sites	Not reported	Nano-TiO_2_	40,000 tonnes/annum	[122]
			Nano-ZnO	1000 tonnes/annum	
			Nano-Ag	40 tonnes/annum	
			Carbon nanotube (CNT)	500 tonnes/annum	
17	MSW collected from the fermentation unit of Istanbul Compost and Recycling Plant in Istanbul, Turkey	Not reported	TiO_2_ and Ag nanoparticles	100 mg/kg	[123]
18	Hamadan city landfill leachate, Iran	Spring season	Iron nanoparticle	2500 mg/L	[124]

## 4. Effect of MSW Landfills on Human Health

**Figure 4:**
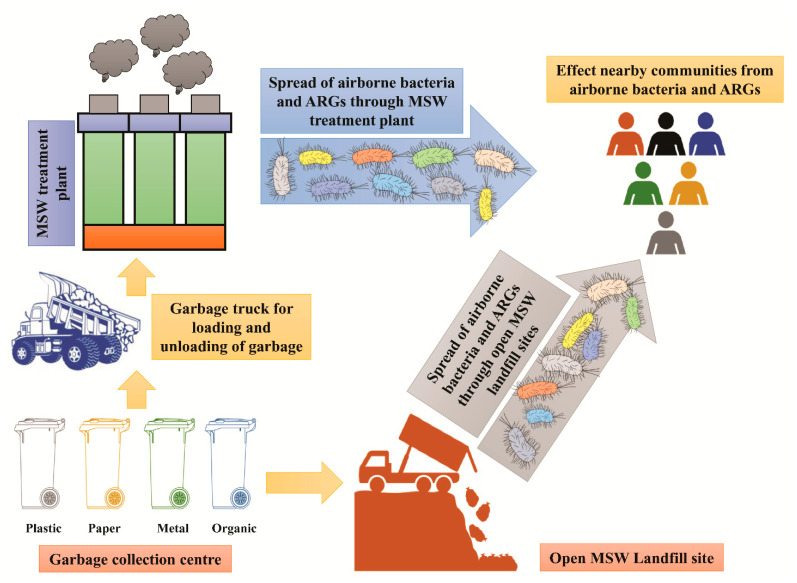
The diagram explains the dissemination of airborne bacterial pathogens during the loading and unloading of garbage vehicles and the spread of ARB from open MSW landfill sites and MSW treatment plants to the nearby human population.

MSW landfills are one of the major resources for microbial air pollution through airborne microorganisms (*Bacillus cereus*, *Staphylococcus lugdunensis*, and *Pseudomonas aeruginosa*) associated with aerosol/bioaerosols [125,126,127]. Bioaerosols are defined as airborne particles of biological origin that consist of bacteria, viruses, fungi, pollen, toxins, allergens, insects, the hair of mammals, products of organisms, and plant parts. The decomposition of waste materials in landfills provides a favourable condition for the formation of bioaerosols and endotoxins and leads to the proliferation of biological agents such as bacteria and fungi. The major health issues or complications related to bioaerosols are acute toxic allergies, respiratory diseases, infectious disease, and cancers [128,129]. Mostly, landfill workers are in direct contact with the landfill area, so these bacterial aerosols have a significant impact on the health of workers with the risk of infectious diseases [130,131,132,133]. Endotoxins are also a major component of bioaerosols, which cause the risk of respiratory diseases. Airborne bacteria originating from MSW landfills need attention to evaluate because most of the airborne bacteria are AR and disseminate ARB and ARG when encountering other environmental bacteria. There is a need to identify the associated microorganisms to bioaerosols and recommend their management strategies. The dissemination of airborne resistant bacteria from landfill sites and MSW treatment plants to nearby human populations is shown in Figure 4.

The extensive use of antibiotics has attracted the attention of risk assessment for human exposure to AR in the environment. Antibiotic medication can change the composition of the gastrointestinal microbiota and influence the emergence of ARB, which could be present in the human gut for a very long time. The imbalance of the human gut microbiota can induce the proliferation of pathogenic bacteria that causes various diseases such as colorectal cancer and intestinal imbalance. Moreover, after the adaptation of AR, the human intestinal bacteria evolve into a superbug that can cause death due to incurability. Landfill sites can contribute to human respiratory diseases, such as asthma and hantavirus pulmonary syndrome, in nearby communities through rodents (rats and mice). Landfills are a hotspot for different organic pollutants, including polychlorinated dibenzo-para-dioxins (PCDDs) and polychlorinated dibenzofurans (PCDFs), that have carcinogenic properties that induce tumour formation in the lungs and skin when contracted by humans and, also, increases the risk of a heart attack [134]. Hydrogen sulphide emissions from landfill sites cannot only harm the respiratory system and contribute to lung diseases such as asthma but can also cause non-respiratory illnesses like diabetes due to exposure to polychlorinated biphenyls. E-waste (electronics and electrical equipment) contains various hazardous and toxic materials, such as lead, zinc, nickel, barium, and chromium, which are directly released into landfill sites or during the recycling process. The unsafe recycling of e-waste in middle- and low-income countries can affect the workers and cause damage to human blood, kidneys, and the central nervous system. Moreover, the study revealed that the effect of landfill pollutants in humans directly depends on the types and duration of the pollutants [135]. Despite various harmful effects, not much research has been conducted recently on the impact of landfill pollutants on the health of closely living human populations. There is a need to identify the associated microorganisms to bioaerosols and recommend their management strategies. Moreover, the presence of medical waste in landfill sites can significantly proliferate and disseminate specific ARGs in different environments; thus, the management of medical wastes is very important. These studies suggest that the improper treatment of hazardous waste and its disposal in landfills could be a potential risk for human health, so it should be necessary to dispose of hazardous waste in landfill sites after proper sanitization or treatment.

## 5. Effect of MSW Landfill Leachate on Soil Health

The open dumping of MSW contains several complex elements, such as heavy metals, humic substances, and degradable and non-degradable organic pollutants that may affect the soil stability and strength due to the percolation of leachate into the soil. It should be noted that groundwater contaminated with leachate is massively used for the irrigation of crops in the adjacent agricultural areas likely to increase the risk of leachate toxicity and, thus, can enter the food chain through vegetation around the site [136]. The generation of toxic gases and contaminated soil can also affect the plants evapotranspiration; infiltration; and uptake of nutrients, metals, and organics [137,138] (Table 3). Therefore, it is hypothesized that any change in the soil physicochemical and biological indicators like the soil microbial biomass and soil microbial enzymatic activities (nutrient transformation and microbial decomposition) may eventually lead to the deterioration of soil composition and health. Therefore, toxicological research focused on the synergic effects of pollutants contained in leachates on soil physicochemical and biological activities (nutrient transformation) are of the utmost importance. The heavy metal pollution in soil emerges due to the mobilization of heavy metal in soil solutions through several solubility reactions of heavy metal ions or compounds and then absorbed by plants or transported into groundwater. Many researches so far have reported that the soil near MSW landfill sites moderately and significantly polluted by heavy metals can adversely affect the nearby vegetation and environmental health [139,140,141]. Therefore, for the improvement of the soil–crop–human ecosystem, it is important to understand the characteristics of the heavy metal pollutants in soil environments contaminated by landfill leachate [39].

Recent studies have noted that the presence of antibiotics in the soil can affect the plant root and soil microbiome, which can also spread the antibiotic-specific ARGs in plant parts [142]. Therefore, it is important to understand the ARGs that transfer from agricultural soil to crop products to human and animal consumption and their effects on human and livestock health. Some microorganisms like *Escherichia, Microbacterium, Stenotrophomonas, Klebsiella, Bacillus*, and *Acinetobacter* isolated from the antibiotic-contaminated area can contribute to the degradation of antibiotics in the soil environment. However, there is a need for additional extensive omics research, including genomics, proteomics, and metabolomics, which can facilitate the deeper evaluation of microbial communities and their antibiotic resistance adaptation and dissemination in soil environments.

**Table 3:** The impact of MSW landfill leachate on the soil physicochemical and microbiological properties.

Sr. No.	Experimental Site	Ecosystem Type/Sample Collection	Parameters Studied	Results	Reference
1	Muskoka Lakes MSW landfill site, Canada	Municipal waste leachate (MWL)	Soil respiration, microbial biomass	The landfill leachate illustrated a negative effect on the soil microbial biomass.	[143]
2	Experimental site of Isfahan University of Technology, Isfahan, Iran	MWL	All essential parameters and other heavy metals	This escalated EDTA (Ethylenediaminetetraacetic acid)-extractable heavy metals in soil. Though it also upsurged the heavy metals concentrations in rice; consequently, crucial use as a liquid fertilizer in soil with more free CaCO_3_. (i.e., calcareous).	[144]
3	Municipal Landfill of the Iasi City, Romania	MWL	BOD (Biological oxygen demand), pH, colour, heavy metals, etc.	BOD, pH, and heavy metal higher concentrations in leachate goes to soil with alarming risks.	[145]
4	Zhouzhou municipal waste (semi-aerobic landfill site) Hebei Province, China	MWL	Bacterial/microbial community structure and methanotrophs analysis through qPCR-DGGE approach, sequences, and phylogenetic analysis	Landfill method differentiation, its age has an impact on bacterial population increase and significantly effects leachate composition.	[146]
5	Experimental station (Site: Instituto Agronômico Do Paraná (IAPAR); Londrina, southern Brazil	MWL	Complete C, N, NH_4_^+^–N, and NO_3_–N. Other important environmental parameters, including heavy metals	Landfill leachate escalated salinizing and N ions; soil physical and microbiological properties remain unchanged.	[147]
6	Visakhapatnam, India	MWL, soil samples were collected from the waste disposal site at various locations. Control soil samples were collected far away from the MSW activity	Estimation of pH, EC, moisture, organic carbon, N, P, and K content	Soil contains a high concentration of the organic compound and heavy metals that may be harmful to plant growth and development.	[148]
7	Field site; Kujawy and Pomorze Province, central Poland	Soil samples were collected from three different landfill locations	TOC, P, K, M, heavy metals (Cu, Zn, Ni, and Pb) estimation, and enzymatic parameters	No influence of heavy metal contaminated soil on dehydrogenases, catalase, alkaline phosphatase, and acid phosphatase activities.	[149]
8	Edebuk Eket Local Government Area, Nigeria	Soil samples were collected from the waste dumpsite as well as away from the waste dumpsite	All essential parameters (viz. pH, EC, C, N, P, K, Ca, Na, Mg, and hardness parameters (Chlorides, Sulphates)	All parameters quantity in high concentration compared to control. Further, dumping in an open site may increase toxic substance that may deteriorate the soil properties.	[150]
9	(Laogang Landfill) Laogang Municipal Waste Disposal Co., Ltd. located at Shanghai, China	MWL, refuse and landfill leachate were collected	NO, N_2_O, and N_2_ gases, the abundance of functional genes	Inhibition of denitrification due to the presence of antibiotics in landfilled refuse (N_2_ production capacity decline under certain conditions).	[151]
10	Zhaozhuang landfill, Jiangsu Province, East China,	MWL	Physicochemical properties, richness and diversity of the microbial community, microbial taxonomic analysis (at the phylum and genus level), correlations analysis	Microorganisms presence as follows: Cover soil (0–30 cm) lowest; stored waste decreased (30–90 cm); 90–150 cm increase. Microbial diversity: high in the top and bottom layers of waste; less in the lower and middle layer of waste.	[152]
11	Asuwei landfill, Changping District, Beijing, China	MWL	Heavy metal analysis, bacterial community structure using 16S rDNA and qPCR-DGGE approach	The effect of dissolved heavy metals on the microbial population in landfill differed from the heavy metals. *Bacteria and actinobacteria*: High at the middle layer *Firmicutes, Proteobacteria, and Actinobacteria:* Very abundant humification effect on Firmicutes. Consequential apprehension of regulation of adjustments of organic matter, heavy metals by microbial communities.	[153]

## 6. Effects on MSW Landfill Leachate on Groundwater and Surface Water Bodies

Groundwater is amongst the most important requirements for better human survival. Municipal solid landfills are considered a hotspot for groundwater and surface water contamination due to the leakage of toxic chemicals [154,155,156]. During heavy rains, the potential penetration of leachate to the surface and groundwater ultimately leads to contamination, which makes the water undrinkable [157]. Many studies have demonstrated the dissemination of landfill contaminants such as phosphate, heavy metals, ammonium, chloride, and sodium to the surface and groundwater [158]. The increased concentration of antibiotics such as ciprofloxacin, β-lactams, vancomycin, cefepime, erythromycin, sulphonamide, and tetracycline in landfill leachate potentially contaminates groundwater resources, which can pose potential risks to public health. The contaminated water sources indicate the contribution of certain ARGs (*sul1* and *sul2*) and MGEs in groundwater contamination, and MGEs play a vital role in the proliferation of ARGs in contaminated groundwater [24]. Furthermore, studies have confirmed that ARGs are supposed to spread in human and domestic animals via anthropogenic activities. The chemical oxidation, reverse osmosis, and photocatalytic methods are used for the removal of residual antibiotics from the groundwater [159]. These methods are comparatively fast in antibiotic degradation, but the working costs are very high. Bioremediation is also one of the most widely used cost-effective and eco-friendly technologies to remove organic and inorganic contaminants from the groundwater through natural microbial populations. A broad variety of microbial populations such as algae (*Cladophora fascicularis, Cladophora* spp., and *Spirulina* spp.); fungi (*Aspergillus versicolor and Aspergillus fumigatus*); bacteria (*Arthrobacter* spp., *Burkholderia* spp., and *Bacillus cereus*); and yeasts (*Saccharomyces cerevisiae* and *Candida utilis*) are used in the bioremediation of heavy metals. A deep understanding of such factors could assist in designing a technique for controlling the contamination of groundwater from hazardous materials. Additionally, the information about the universal marker for ARG identification and the influence of environmental factors on the dissemination of ARGs is necessary to understand the fate of ARB in groundwater environments.

## 7. Conclusions and Future Perspectives

The worldwide exponential growth in the human population has led to increased urbanization, hospitalization, and industrialization that can facilitate the generation of massive quantities of MSW. The rapid increase in MSW generation is a real threat to global human health and is one of the major challenges to the environment. In terms of the economy, the exponential rise in MSW is also imposing a high-cost burden on the municipal budget annually. In the present scenario, landfill leachate is one of the major hotspots for ARB, ARGs, MGE, ECs, and organic and inorganic pollutants and is responsible for dissemination into nearby natural environments. The anthropogenic activities may be involved in the dissemination of contaminants from anthropogenic to natural environments. Although many studies and reports proved this phenomenon, the scope and epidemiological studies are not well-understood. This review summed up the influence and effect of MSW landfill leachate on surface and groundwater resources, soil quality, and, also, on human health. It is highly recommended to make policies and improve landfill sites for the betterment of nearby contaminated ecosystems and communities. The upgrade of open landfill sites should be covered to control the leachate production and its drain into the groundwater and soil around the landfill. The present review strongly suggests that future research should be focused on the impact of anthropogenic activities to better understand the dynamics and dissemination of ARGs from landfill leachate into natural environments. There is a need for the advancement of the present technology in landfill treatment plants for the removal of ARB, ARGs, and ECs and to develop the appropriate policy to reduce the diverse risk associated with the ARG level and improve the surrounding environment with proper management. The current status and knowledge regarding the ongoing threat of AR in the context of human health and the renowned sparking hypothesis of O’Neill [160] that resistance to antibiotics can be a major problem by 2050 demand the establishment of a new system of collaboration between national and international societies, including nongovernmental organizations (NGOs), to make new strategies and laws to encounter AR based on the “One Health” notion.

## Figures and Tables

**Table 1:** Detection of antibiotic resistance genes (ARGs) and metal resistance genes (MGEs) in the leachate samples of different landfill sites.

Sr. No.	Landfill Location	Sampling Date	Capacity	Heavy Metal	Antibiotics Measured	ARGs and MGEs Analyzed	Remark	References
1	Shanghai, China	May, 2015	10,000 tonnes/day	Lead, Zinc, Copper, Arsenic, Cadmium, Chromium, Cobalt, Manganese, and Nickel	Sulfadiazine (C_10_H_10_N_4_O_2_S), Sulfamerazine (C_11_H_12_N_4_O_2_S), Sulfapyridine (C_11_H_11_N_3_O_2_S), Sulfathiazole (C_9_H_9_N_3_O_2_S_2_), Sulfaquinoxaline (C_14_H_12_N_4_O_2_S), Sulfamethoxazole (C_10_H_11_N_3_O_3_S), and Sulfamethazine (C_12_H_14_N_4_O_2_S)	Sulfonamide ARGs: sul-I, II, III; Integrons: IntI-1, 2, 3; Insertion sequences: IS-26, IS-CR3; Plasmids: tra-A, trb-C; Transposons: mer-A, tnp-A/Tn21.	The study concluded that the ARGs were increased with the age of landfill and MGEs were also highly abundant in the landfills and highly correlated with ARGs.	[56]
2	Xiamen, China	-	3900 tonnes/day	-	-	285 ARGs and 10 MGEs	This study indicated that the landfill treatment plant cannot effective in the removal of the ARGs except tetracycline and not even able to stop the shifting of resistome and microbial community to other downstream environments. They suggested that there is a need to optimize the treatment process and should improve the ARGs removal efficiency.	[22]
3	Shanxi Province, China	June, 2016	-	Arsenic, Chromium, Cadmium, Copper, Nickel, Lead, and Zinc	-	Tetracycline ARGs: *tet*A, *tet*A/P, *tet*B/P *tet*C, *tet*E, *tet*K, *tet*L, *tet*G, *tet*M, *tet*O, *tet*Q, *tet*S, *tet*T, *tet*W. β lactams ARGs: *bla_CTXM_*, *bla_TEM_*, *bla_SHV_*_,_ and *bla_ampC._* Sulfonamide ARGs: *sul*I, *sul*II, and *sul*III; Fluoroquinolone ARGs*: qnr*A, *qnr*B, *qnr*S; Macrolide ARGs: *ere*A, *ere*B, *mph*A; Class-1 integrons: *int*I1; Transposons: *tnp*A.	The study examined the Metal resistant genes and ARGs were present in the differentially metal polluted sites. Arsenic (arsC) and macrolide resistant genes (ereA) were highly abundant.	[60]
4	Zhejiang Province, China	July to November 2018	-	Lead, Zinc, Copper, Cadmium, Chromium, and Nickel	21 antibiotics from three different groups: Fluoroquinolones, Macrolides, and Sulfonamides	Fluoroquinolone ARGs: qnrS, mexF, qnrD. Macrolide ARGs: ermA, ermB, mefA. Sulfonamides ARGs: sul1, sul2.	Most of the landfills sites were highly ARGs contaminated with fluoroquinolone macrolide and sulfonamide resistant genes but these ARGs were not showing correlation with the corresponding antibiotics. The role of MGEs was very significant in the ARGs abundance.	[61]
5	Southwest China: Jiangcungou Landfill and Guiyang Gaoyan Landfill; Southwest China: Guiyang Biliba Landfill;East China: Suzhou Qizishan Landfill and Hangzhou Tianzilin Landfill	November 2018 to January 2019	-	Iron, Copper, Zinc, Arsenic, Chromium, Cadmium, Lead, Manganese, Nickel, Antimony, and Cobalt	Tetracycline: oxytetracycline, doxycycline; Sulfonamides: sulfadiazine, sulfamethazine, sulfamethoxazole, Fluoroquinolones: trimethoprim, norfloxacin, ofloxacin, pefloxacin, enrofloxacin; Macrolides: erythromycin-H2O and roxithromycin; β-lactams: amoxicillin, cefalexin.	Sulfonamides ARGs: sul1, sul2 Macrolides ARGs: ermB, mefA Tetracyclines ARGs: tetM, tetQ β-lactams ARGs: blaCTXMMultidrug-resistant subtype: mexF Integron: intl1	During the landfilling process antibiotics, heavy metals and ARGs are significantly distributed in the landfills. Highly dominant ARGs investigated in this study were sul1, ermB, and sul2.	[62]
6	Laogang (LG) Landfill Shanghai, China	-	13,000 tonnes/day	-	-	Sulfonamides ARGs: sul1, sul2 and β-lactams ARGs: blaOXA, blaCTX-M, blaTEM	This research generated a few models which explained the role of integrons and environment factors that drastically influence the level of ARGs in landfill leachate.	[63]
7	Laogang (LG) Landfill Shanghai, China	June and January 2016	13,000 tonnes/day	-	-	Sulfonamides ARGs: sul1, sul2 Macrolides ARGs: ermB, mefA β-lactams ARGs: blaOXA, blaCTX-M, blaTEM, and blaNDM-1 Integrons: intl1 and intl2	The study found out the occurrence of ARGs associated with ARB were very high during the treatment process and were disseminated into the downstream environment.	[64]
8	Qicungou Landfill, northwest China; Gaoyan Landfill, southwest China; Shuige Landfill; Qizishan Landfill; Laogang Landfill; Tianzilin Landfill, east China; Xiaping Landfill, south China	-	5500 tonnes/day; 2200 tonnes/day; 3000 tonnes/day; 2500 tonnes/day; 10,000 tonnes/day; 6000 tonnes/day; 4500 tonnes/day	Cadmium, Chromium, Copper, Manganese, Nickel, Lead, and Zinc	Ofloxacin, norfloxacin, enrofloxacin, and pefloxacin, cephalosporin, and amoxicillin	Fluoroquinolone ARGs: qnrA, qnrB, qnrD, qnrS. β-lactams ARGs: blaCTX-M, blaOXA10, blaOXY, blaPER, blaSFO, blaSHV, penA, ampC. Transposase: tnp01, tnpA02, tnpA03 Integron: intl1, intl2	The content of fluoroquinolone and β-lactams ARGs in leachates were found very high, across China and the close association of these genes with MGEs could help in the dissemination of ARGs in the different environment via landfill leachate.	[65]
9	Guangzhou, China	-	6500 tonnes/day	Copper, Zinc, Nickel, and Chromium	-	Tetracyclines ARGs: tetM, text, tetA, tetB, tetC, tetL, tetQ, tetS, tetW Sulfonamide ARGs: sul1, sul2, AmpC β-lactamase ARGs: EBC, FOX, Integron: intI1	The study indicated that the abundance of ARGs level in leachate partially depended on heavy metals.	[66]
10	Xi’an, China	-	5500 tonnes/day	-	-	sulI and tetO	This study found that the level of sulI and tetO ARGs were decreasing with the age of landfill.	[48]
11	Hulin, Xupu and landfill reservoir, Shanghai	April and July 2014	1700 tonnes/day	Lead, Zinc, Copper, Arsenic, Cadmium, Chromium, and Nickel	Sulfonamide, quinolone, tetracycline, macrolide, and chloramphenicol	Sulfonamide ARGs: sul1, sul2. Tetracycline ARGs: tetQ, tetM. Macrolide ARGs: ermB, mefA	In this study most of the measured ARGs significantly associated with the Cd and Cr heavy metals.	[67]
12	Shenzhen: Laohukeng landfill; Taiyuan: Houcun landfill; Xi’an: Jiangchungou landfill; Tangshan: Jianzigu landfill; Shanghai: Laogang landfill; Yongchuan: Yongchuan landfill	8 Sept., 12 Sept., 15 Sept., 20 Sept., 28 Sept., 8 Nov., 2012	-	-	-	Tetracycline: tetO, tetW.β-Lactam: blaTEM. Sulfonamide: sulI, sulII	This study investigated that the tetracycline and sulfonamide resistance genes copy numbers were significantly correlated with the 16S rRNA copy number of environmental bacteria. This result notably showed that the bacterial species of landfill leachate may play a vital role in the horizontal transfer of ARGs.	[14]

## Data Availability

This is a review article all the data have been taken/retrived from the cited references.

## Abbreviations

MSW	Municipal solid waste
MWL	Municipal waste leachate
ARB	Antibiotic resistant bacteria
ARGs	Antibiotic resistance genes
MRGs	Metal resistance genes
AR	Antibiotic resistance
AMR	Antimicrobial resistance
HGT	Horizontal gene transfer
MGEs	Mobile genetic elements
WWTPs	Wastewater treatment plants
PPCPs	Pharmaceuticals and personal care products
EDCs	Endocrine disrupting chemicals
WHO	World Health Organization
COD	Chemical oxygen demand
PFAS	Polyfluoroalkyl substances
PFAA	Perfluoroalkyl acids
ECs	Emerging contaminants
